# A Pilot Randomized Controlled Trial of a Web-Based Growth Mindset Intervention to Enhance the Effectiveness of a Smartphone App for Smoking Cessation

**DOI:** 10.2196/14602

**Published:** 2019-07-09

**Authors:** Vasundhara Sridharan, Yuichi Shoda, Jaimee Heffner, Jonathan Bricker

**Affiliations:** 1 University of Washington Seattle, WA United States; 2 Fred Hutchinson Cancer Research Center Seattle, WA United States

**Keywords:** addictive behavior, smoking behaviors, smoking cessation, health technology, mobile apps, psychological theory

## Abstract

**Background:**

Although smartphone apps have shown promise for smoking cessation, there is a need to enhance their low engagement rates. This study evaluated the application of the growth mindset theory, which has demonstrated the potential to improve persistence in behavior change in other domains, as a means to improve engagement and cessation.

**Objective:**

This study aimed to explore the feasibility, utility, and efficacy of a Web-based growth mindset intervention for addiction when used alongside a smoking cessation app.

**Methods:**

Daily smokers (N=398) were all recruited on the Web and randomly assigned to receive either a cessation app alone or the app plus a Web-delivered growth mindset intervention. The primary outcome was engagement, that is, the number of log-ins to the smoking cessation app. The secondary outcome was 30-day point prevalence abstinence at 2-month follow-up collected through a Web-based survey.

**Results:**

The 2-month outcome data retention rate was 91.5% (364/398). In addition, 77.9% (310/398) of the participants in the experimental arm viewed at least 1 page of their growth mindset intervention, and 21.1% (84/398) of the group viewed all the growth mindset intervention. The intention-to-treat analysis did not show statistically significant differences between the experimental and comparison arms on log-ins to the app (19.46 vs 21.61; *P*=.38). The experimental arm had cessation rates, which trended higher than the comparison arm (17% vs 13%; *P*=.10). The modified intent-to-treat analysis, including only participants who used their assigned intervention at least once (n=115 in experimental group and n=151 in the control group), showed that the experimental arm had a similar number of log-ins (32.31 vs 28.48; *P*=.55) but significantly higher cessation rates (21% vs 13%; *P*=.03) than the comparison arm.

**Conclusions:**

A growth mindset intervention for addiction did not increase engagement rates, although it may increase cessation rates when used alongside a smartphone app for smoking cessation. Future research is required to refine the intervention and assess efficacy with long-term follow-up to evaluate the efficacy of the mindset intervention.

**Trial Registration:**

ClinicalTrials.gov NCT03174730; https://clinicaltrials.gov/ct2/show/NCT03174730

## Introduction

### Background

Cigarette smoking is the leading cause of preventable death and disease in the United States [1]. To reduce the public health burden of smoking, there is an ongoing need for low-cost, high reach public health interventions for tobacco cessation [1]. In response to this need, smartphone app–based smoking cessation interventions have become increasingly prevalent [2]. Although this area of research is still nascent, clinical trials evaluating smoking cessation apps have shown that apps such as *Smokefree28* [3], *SmartQuit* [4,5], *Clickotine* [6], and other apps [7] have yielded promising quit rates over 2 to 6 months. Although app-based interventions are promising because of their high potential reach and low cost, smokers’ engagement with these interventions remains low, and there is much room for improving their efficacy [3,8]. Increased engagement with apps is a key target for improving efficacy because smokers who engage more tend to have higher quit rates [6,8]. Overall, there is a need for theory-based approaches to improve the efficacy of existing cessation apps. This study evaluated the application of the growth mindset theory [9], which has demonstrated the potential to improve persistence in behavior change in other domains, as a means to improve engagement and cessation.

### Application of the Growth Mindset Theory to Engagement

People develop lay theories about the nature of human attributes such as intelligence and personality [10]. These lay theories, also referred to as fixed and growth mindsets, are fundamental belief systems about the malleability of different human attributes. A person holding a *fixed mindset* about an attribute (eg, intelligence or addiction) considers that it is a permanent entity that is firmly entrenched in an individual’s personality. Contrasting this, a person holding a *growth mindset* about that attribute believes that the attribute is malleable [10].

A belief system about the malleability of addiction is referred to as an addiction mindset [11]. A person can have a *fixed mindset* about addiction, in which they believe that addiction is a permanent attribute of a person and cannot change. Alternately, they can have a *growth mindset* about addiction, whereby they believe that addiction is changeable. Survey research suggests that smokers with a growth mindset about addiction to cigarettes (nicotine) tend to be more motivated and willing to persist with quitting [11]. In addition, as the literature suggests that a mindset is particularly effective at changing behavior by improving participants’ persistence in goal-oriented behavior [12], the addiction mindset was chosen as a possible target for an intervention to improve both engagement with apps and cessation.

Experimental research has shown that interventions fostering a growth mindset show promise for behavior change. In educational contexts, interventions fostering a growth mindset of intelligence have been an effective way to improve academic performance in students [13-15]. Growth mindset interventions have been applied in other domains including reducing aggressive behavior [16], reducing stress, and improving coping behaviors [17]. Growth mindset interventions have also been beneficial for improving health behaviors in both young and adult groups, including preventing weight gain among overweight participants [18] and improving mental health [19]. Despite the promise of changing mindsets to change behavior, no work to date has explored the application of this theory to interventions for addictive behavior.

### This Study

The goal of this study was to evaluate a growth mindset intervention for improving engagement with and effectiveness of an established smoking cessation app (*SmartQuit*) for adult daily smokers. The SmartQuit app was ideal for this study because its effectiveness for engagement and cessation has been reported in 2 clinical trials [4-5]. Furthermore, engagement with different features of SmartQuit and engagement patterns associated with successful cessation have been identified in previous research [8,20]. This study evaluated the addition of a growth mindset of addiction (to nicotine) component by randomly assigning adult current smokers to either SmartQuit plus a Web-delivered growth mindset intervention or a comparison arm (only SmartQuit). The intervention was specific to addiction to nicotine, referring only to cigarette smoking. The primary outcome measure was engagement with SmartQuit, and the secondary outcome was cessation.

## Methods

### Design and Randomization

Participants were randomized (1:1) to either the experimental group (growth mindset intervention+SmartQuit app, n=199) or the control group (SmartQuit app only, n=199) using randomly permuted block randomization, stratified by heavy daily smoking (yes or no to 20 cigarettes per day or more) and education (yes or no to high school or less), as these are common predictors of cessation [21,22]. The growth mindset intervention was delivered through an emailed link to a website. Participants were blinded to the exact nature and conditions in the study (see section *Blinding*) to minimize any potential placebo effect of 1 group receiving an additional intervention. The study was registered on ClinicalTrials.gov (NCT03174730).

### Recruitment

#### Eligibility Criteria

The eligibility criteria were as follows: (1) aged ≥18 years, (2) smoked ≥5 cigarettes per day for the past 12 months, (3) ready to quit in the next 30 days, (4) lived in the United States and planned to remain for next 3 months, (5) could read English, (6) had access to a smartphone (running iOS version 8 or higher or running Android version 4.4 or higher) and could download an app, (7) had access to the internet and personal email, (8) not currently enrolled in other smoking cessation treatment, (9) never participated in previous studies by the same research group, and (10) willing to be randomized to treatment and willing to complete surveys at baseline and follow-up.

#### Sample Size

Consistent with the aims of this pilot trial, the sample size was determined using a precision-based approach [23] with the main outcome of engagement with the smoking cessation app. Using available preliminary data on SmartQuit app log-ins [4,5], a sample size of 300 was determined to provide 80% power to detect the differences in number of log-ins between study arms. A threshold minimum effect size of Cohen d=0.2 was used to provide precision toward estimating the engagement effects in a large phase 3 trial. Although the target sample was 300, the sample size was increased to 398 after 2 months of recruitment to account for data loss from participants not accessing SmartQuit (27% of the first 150 participants). See section *Implications for Engagement* for further detail.

#### Participants and Enrollment

**Figure 1:**
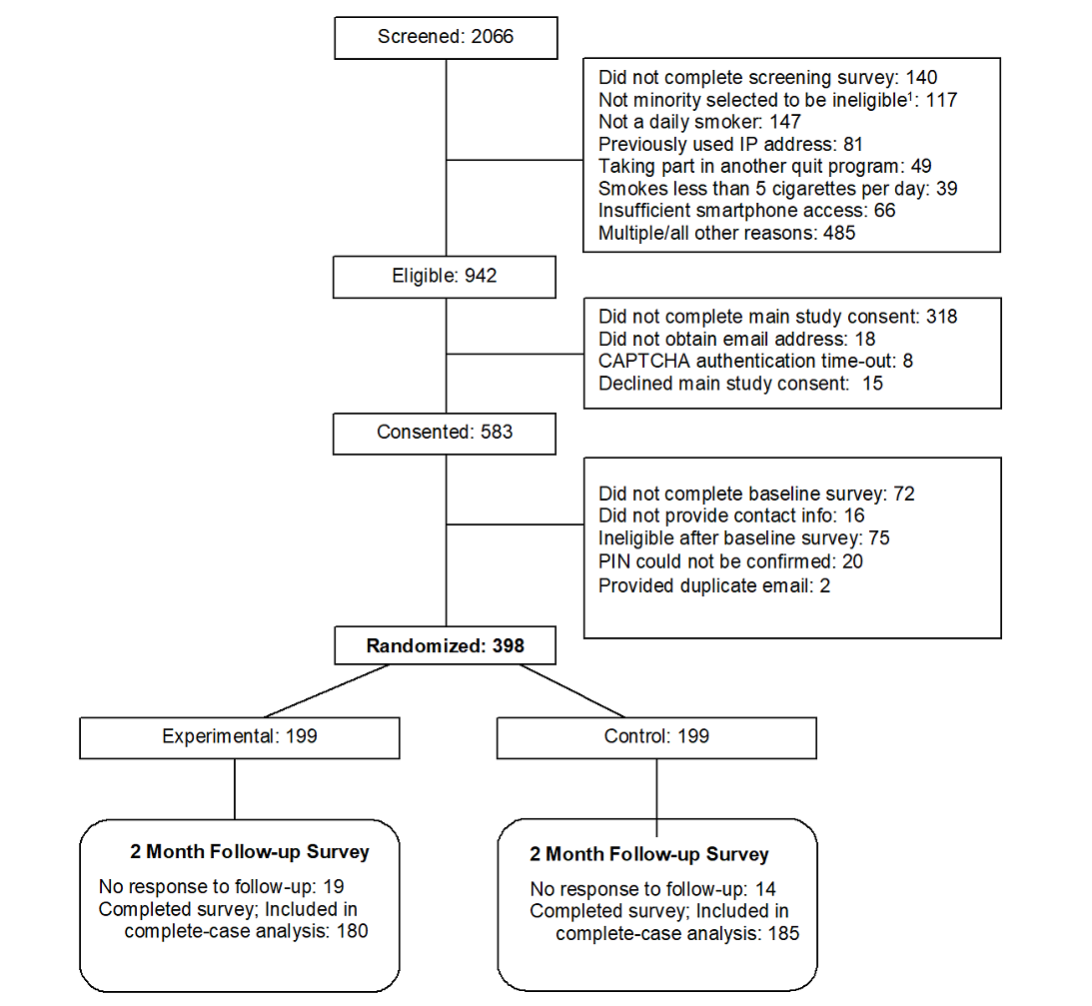
Participant flow diagram. To increase the enrollment of racial and ethnic minorities, some nonminorities who were otherwise eligible for study enrollment were randomly selected to be excluded. IP: Internet Protocol; PIN: personal identification number.

Adult smokers (N=398) were recruited between June and October 2017. Figure 1 shows the study participant flow diagram (Consolidated Standards of Reporting Trials). Recruitment strategies included the use of an internet survey panel and Facebook advertisements to recruit a national sample. Targeted advertisements and website content were used to ensure that the sample reached the minimum 25% male and the minimum 25% minority enrollment targets. A minimum inclusion level for men was added to recruitment criteria because studies using similar recruitment methods tend to overrecruit women [24]. Potential participants completed a Web-based screening survey to assess eligibility. If they screened eligible, they provided Web-based consent and completed a baseline survey and a contact form. Enrollment fraud deterrence included CAPTCHA authentication and review of internet protocol addresses for duplicates or non-US origin participants.

#### Blinding

The study was presented to participants as *a research study comparing 2 technology-based quit-smoking programs* to maintain blinding of treatment group assignment. Neither research staff nor study participants had access to randomized study arm assignments. Participants were debriefed at the end of the study with the full purpose and differences between groups in the study.

### Follow-Up Assessment

Participants completed a follow-up survey at 2 months after randomization. Moreover, 2 weeks before the survey, participants received US $2 as a preincentive and a letter notifying them to expect the survey. Participants received US $25 for completing the survey and an additional US $10 bonus if they completed the Web-based survey within 24 hours of receiving the invitation. Participants who did not complete the Web-based survey within 18 days were sequentially offered opportunities to do so by phone and a mailed survey. Further noncompleters were finally sent a postcard after 30 days with the option to provide their smoking status. Details of our recruitment and retention techniques used in this study are identical to our other electronic health (eHealth) studies described elsewhere [24]. This assessment method yielded a 91.5% (364/398) follow-up rate at 2 months, with 65.9% (240/364) of those responding within 24 hours of survey receipt.

### Description of the Growth Mindset Intervention: MIND Tips

The theoretical basis used for all the materials in the MIND tips was the mindset theory [9]. The goal of the intervention was to influence participants’ beliefs about the permanence of addiction. Toward this goal, the first step was to identify the specific aspects of addiction that are considered permanent. The literature on nicotine and tobacco addiction was reviewed, and *permanence belief* themes were extracted qualitatively from the review process by all authors (see Multimedia Appendix 1 for additional references). These were refined into 6 specific beliefs with input from subject matter experts (additional scientists in tobacco treatment and counselors). The review was limited to nicotine/tobacco addiction and not expanded to addiction in general. The final 6 beliefs used for creating the intervention content included the following: *addiction is permanent because it is genetic*, *some people will always be addicted because they have an addictive personality*, *addiction is permanent because it irreversibly changes the brain*, *addiction is permanent because withdrawal symptoms may persist after cessation*, *addiction is permanent because people can feel like smoking even years after quitting*, and *failure to quit smoking is indicative of a permanent habit*.

Next, 6 packets of information called MIND Tips (MIND is an acronym for Mindset Intervention for Nicotine Dependence) were created by the authors of this paper in the form of Web-based lessons to counteract each of these 6 beliefs. Moreover, 2 additional lessons were created to provide an introduction and summary for the program. Similar to growth mindset interventions in education and weight loss [13,18], these lessons were constructed to provide scientific evidence for the capacity to change addiction. For example, the lesson discussing genes for addiction explained that having specific genes does not guarantee that a person will always be addicted [25,26]. Furthermore, every tip featured both a testimonial of a (fictional) former smoker demonstrating a growth mindset, consistent with recommendations about their persuasiveness from health communication research [27]. An example of a testimonial from the study showed a person’s struggle with their belief in an addictive personality (an intrinsic aspect that cannot change or a fixed mindset of addiction). After presenting scientific information about personality, their quote changes to say that learning this new information showed that personality does not prevent successful quitting, and anyone can quit (growth mindset). The quote is as follows:

I’ve always liked trying new things just for the experience. Unfortunately, the one thing I tried and could not stop was cigarettes. When I struggled to quit, my mom said I’ve always had an addictive personality. After learning that my personality does not matter for quitting, I was able to kick the habit for good. It wasn’t easy, but it was worth all the effort because it feels so good to be free at last.

A user-centered design process for psychological interventions [28] was used to create and test the MIND content. To make it user-friendly, each lesson was 300 to 500 words long and was at an eighth grade or lower reading level. Remote user testing with 25 current daily smokers for *each* lesson tested the acceptability, usability, and feasibility of remote administration of the MIND content.

These 8 lessons were delivered to participants via an emailed link to a website in the experimental group. Participants in the intervention group received their first MIND Tip email (containing the link of the first lesson) on the same day that they enrolled. They did not have to complete reading it to receive the following content or gain access to SmartQuit. The remaining lessons were sent, one at a time, every 3 days in a preset order (introduction, withdrawal, genes, changes in the brain, personality, urges and cravings, failure to quit, and summary). In this way, the MIND Tips were spaced out over 24 days to prevent overloading participants with content at the time of enrollment. When participants finished reading each lesson, they were provided a link to download the PDF file of the lesson if they wished. The links to the MIND content were active from the day they were pushed out until the end of the study period (2-month window for each participant). Participants could read the MIND Tips content any time during this window and as frequently as they wished.

### Description of the SmartQuit Program

Participants in both groups received access to SmartQuit, a smartphone app created to facilitate smoking cessation [29]. As soon as they were confirmed enrolled in the study, participants in both groups were provided a log-in and password to open the app by email. Once they logged in, the app helped them create a quit plan, set a quit date, and establish why they wish to quit. Full details of the app are described elsewhere [4,5,8]. Participants were informed they could use the app as they wished for 2 months.

### Measures

#### Demographics and Smoking History

Participants reported demographic information including their age, gender, race and ethnicity, sexual orientation, education, and employment. Participants who were marked eligible in a short screener were provided up to a week to complete the baseline measures online. Participants reported the number of cigarettes they smoked per day in the past 30 days, as well as the number of years they have been a regular smoker, and the number of quit attempts in the past 12 months. They also completed the Fagerström Test of Nicotine Dependence (FTND) [30].

#### Mindset and Motivational Variables

Participants completed, at baseline and 2-month follow up, the 6-item Addiction Mindset Scale (AMS; baseline alpha=.68 and follow-up alpha=.73) [11]. The AMS consists of 6 statements that measure beliefs that addiction is permanent (eg, *a person’s addiction can never fully leave them*) on a strongly agree to strongly disagree scale. Participants received instructions to consider only addiction to nicotine (ie, from cigarette smoking) only for all statements. Participants completed the 8-item measure of motivation using the Commitment to Quitting Smoking Scale [31], which assesses their willingness to persist in staying quit despite discomfort or other difficulties at both baseline (alpha=.91) and follow-up (alpha=.93). As a measure of self-efficacy for quitting smoking, participants reported their confidence in staying abstinent at the 2-month follow-up using an adapted single item, “On a 0-100 scale, where 0 is not at all and 100 is extremely confident, how confident are you that you will be abstinent 2 months later?” [32].

#### Utilization of the Growth Mindset Intervention

Objective measures of website utilization were tracked through server-recorded, time-stamped page views.

#### Outcome Measures: App Engagement

Participants’ use of the SmartQuit app was automatically recorded for the duration of the study, that is, 2 months from the date of enrollment for each participant. The main indicator of use recorded for this study was the number of log-ins. Additional metrics of interest included the number of days of use and whether participants received the certificate of completion awarded inside the app (participants who completed a quit plan, viewed the 8 main exercises, used the urge tracking feature, and viewed additional content from a help menu received this certificate), which predicted 4 times higher odds of cessation in a previous trial [8].

#### Outcome Measures: Cessation and Smoking Behavior

Two months after the date of randomization, participants completed the FTND and reported the last time they smoked a cigarette, how many cigarettes per day they smoked on average in the last 30 days, and how many quit attempts they made. Cessation was defined as self-reported 30-day point prevalence abstinence (PPA; ie, no smoking at all in the past 30 days) at the 2-month follow-up. Biochemical validation of quit status was not used in this study. However, research suggests that biochemical verification is not required in studies where data are collected through Web or mail without any face-to-face contact and which present limited demand characteristics [33,34].

### Statistical Analysis

All analyses were conducted with the analysis software R Studio version 3.4.0 [35]. For all analyses, a significance level of .05 was used. Differences between demographic variables across arms at baseline were examined using *t* tests and Fisher exact tests for continuous and categorical variables [36,37]. If any baseline variables were found imbalanced across arms, *and* these variables were predictive of the outcome of the analysis, they were included as covariates in analyses comparing study arms [38]. Logistic regression models were used to examine differences in cessation between groups. The primary analysis method was a complete case analysis with the intent-to-treat sample, which covers 91.5% (364/398) of the recruited sample. A secondary sensitivity analysis was included, with missing cessation data coded as smoking to allow comparison with other trials [39]. In addition, a modified intent-to-treat analysis was conducted on the sample that accessed their assigned intervention materials at least once. This includes only those participants who had logged in at least once to SmartQuit (both arms) and had viewed at least 1 page (out of the 8 page views required for complete adherence) of the MIND content (experimental arm only; n=266).

Negative binomial regression models were used for predicting 2 engagement outcomes (number of log-ins and number of days logged in) to account for zero inflated distributions [40]. All analyses exploring group differences between the control and experimental groups controlled for the randomization factors of education and heavy smoking to avoid loss of power [41]. Further covariates were included only if they were significantly and independently associated with both the predictor and outcome variables in models [42].

## Results

### Description of Sample

Table 1 shows the demographics of the sample across the intervention and control arms. The only significant difference in characteristics at baseline was self-efficacy to quit. The control group had significantly higher baseline self-efficacy to abstain from smoking (*P*=.002). As self-efficacy is predictive of smoking cessation in this study (*P*<.001), models predicting cessation from group assignment controlled for baseline self-efficacy.

**Table 1:** Baseline demographics, self-reported mental health, smoking history, and behavior of participants in the Mindset Intervention for Nicotine Dependence study for the intention-to-treat sample.

Characteristics		Total (N=398)	Control (n=199)	Intervention (n=199)	*P* value^a^
**Demographics**					
	Age (years), mean (SD)	42.0 (12.3)	42.0 (12.5)	42.1 (12.0)	.88
	Male, n (%)	165 (41)	84 (42)	81 (41)	.84
	Caucasian, n (%)	309 (79)	157 (79)	152 (78)	.99
	African American, n (%)	60 (15)	32 (16)	28 (14)	.75
	Asian, n (%)	1 (<1)	1 (<1)	0 (0)	>.99
	Native American or Alaska Native, n (%)	3 (1)	1 (<1)	2 (1)	.98
	Native Hawaiian or Pacific Islander, n (%)	1 (<1)	0 (0)	1 (<1)	.99
	More than 1 race, n (%)	19 (5)	8 (4)	11 (6)	.60
	Hispanic, n (%)	45 (11)	20 (10)	25 (13)	.53
	Married, n (%)	125 (31)	57 (29)	68 (34)	.28
	Working, n (%)	195 (49)	99 (50)	96 (48)	.84
	High school or less education, n (%)	156 (39)	78 (39)	78 (39)	>.99
	Lesbian, gay, or bisexual, n (%)	68 (17)	39 (20)	29 (15)	.23
**Self-reported mental health, n (%)**					
	Anxiety disorder	146 (37)	77 (39)	69 (35)	.47
	Depression	144 (36)	76 (38)	68 (34)	.47
	Bipolar disorder	56 (14)	30 (15)	26 (13)	.67
	Schizophrenia	9 (2)	3 (2)	6 (3)	.50
	Alcohol abuse	16 (4)	7 (4)	9 (5)	.80
	Drug abuse	24 (6)	9 (5)	15 (8)	.29
	No mental health conditions	199 (50)	94 (47)	105 (53)	.32
**Smoking behavior**					
	Fagerström Test for Nicotine Dependence score, mean (SD)	5.84 (2.1)	5.87 (2.1)	5.82 (2.0)	.81
	High nicotine dependence, n (%)	234 (59)	117 (59)	117 (59)	>.99
	Cigarettes per day, mean (SD)	19.0 (16.2)	19.0 (16.5)	19.1 (15.9)	.94
	Smokes more than half pack per day, n (%)	279 (70)	137 (69)	142 (71)	.66
	Smokes more than 1 pack per day, n (%)	84 (12)	43 (22)	41 (21)	.90
	Used electronic cigarettes at least once in past month, n (%)	85 (21)	37 (19)	48 (24)	.22
	Quit attempts in the past 12 months, mean (SD)	1.0 (2.5)	0.8 (2.0)	1.1 (2.9)	.33
	Self-efficacy, mean (SD)	71.6 (22.6)	75.2 (21.2)	68.0 (23.4)	.002
	Commitment to quitting, mean (SD)	4.00 (0.7)	4.04 (0.7)	3.96 (0.7)	.31
**Friend and partner smoking**					
	Close friends who smoke, mean (SD)	2.5 (1.8)	2.5 (1.8)	2.4 (1.8)	.48
	Number of adults in home who smoke, mean (SD)	1.5 (0.8)	1.6 (0.9)	1.5 (0.8)	.27
	Living with partner who smokes, n (%)	136 (34)	63 (32)	73 (37)	.34
**Theory-based measures**					
	Addiction Mindset Scale score, mean (SD)	3.33 (0.8)	3.31 (0.8)	3.35 (0.8)	.69

^a^*P* values are reported for *t* tests (for continuous variables) and Fisher exact tests (for categorical variables) comparing demographics across groups.

### Intention-to-Treat Analyses

#### Treatment Adherence and Change in Mindset

In the intervention group, 78.4% (156/199) of the participants viewed at least 1 page of the MIND tips, and 21.1% (42/199) of the group viewed all 8 tips. On average, participants in the intervention group viewed 4.16 (SD 3.38) tips out of 8 total tips. Controlling for the baseline level of growth mindset, the intervention group’s mean AMS score after 2 months (3.42 [SD 0.90]) was not significantly different from the control group (3.35 [SD 0.93]; *B*=0.05; 95% CI −0.13 to 0.22; *P*=.59). We also found no group-level differences between AMS score at follow-up (control group 3.35 [SD 0.93] vs intervention group 3.42 [SD 0.90]; *P*=.46).

#### Engagement: Primary Outcome

The results are shown in Table 2. Across both groups, 72% of the participants logged in at least once to the SmartQuit app. Participants in the control arm logged in an average of 21.61 (SD 37.74) times (median 6.00; interquartile range [IQR] 25.50) and in the experimental arm, 19.46 (SD 30.20) times (median 5.00; IQR 25.00). Participants in the experimental arm did not log in to the app more than control arm participants (*P*=.38).

#### Engagement: Additional Metrics of Interest

Intervention group participants logged in for an average of 11.73 days (median 4.00; IQR 17.5) compared with the control group’s 12.19 days (median 5.00; IQR 5.00), and this difference was not significant (*P*=.97). The proportion of participants receiving certificates of completion was highly similar in the intervention (31%) and control (30%) groups (*P*=.74).

#### Cessation: Secondary Outcome

In complete case analysis, the 30-day PPA rates at 2-month follow-up in the experimental and control conditions were 17% and 14%, respectively (odds ratio [OR] 1.64; 95% CI 0.90-3.00; *P*=.10), representing a 64% increase in the odds of quitting in the experimental group relative to the control group. The results did not change when missing data were treated as smoking (OR 1.54, 95% CI 0.88-2.76; *P*=.14).

#### Cessation: Progress Metrics

For cessation progress, the smokers in the intervention group reported nonsignificant decreases in smoking (mean decrease in number of cigarettes per day=4.66 vs 3.01, *B*=−1.90; *P*=.07;) and marginal reduced dependence as measured by the FTND score (mean score decrease=1.55 vs 1.11; *B=* −.53; *P*=.05) compared with participants in the control group.

#### Modified Intention-to-Treat Analyses

Results for modified intention-to-treat (ie, participants with ≥1 SmartQuit log-in for both arms and at least 1 page view of the 58 total pages of MIND content for participants in the experimental arm) are summarized in Table 3. When examining only the participants included in this analysis, the intervention group (n=115) did not differ from the control group (N=151) in number of log-ins to the SmartQuit app (M=32.31 vs 28.48; *P*=.55). Descriptively, the participants in the intervention group tended to log in more days (mean 20.10 days) compared with the control group (mean 15.46 days; *P*=.06) and were more likely to receive the certificate of completion in SmartQuit (50% vs 38%; *P*=.07) although these differences were nonsignificant.

**Table 2:** Smoking cessation and engagement with cessation program at 2-month follow-up. Results are adjusted for 2 stratification factors (heavy smoking and education). Cessation results are adjusted for baseline self-efficacy.

Outcome variable		Overall (N=398)	Control (n=199)	Intervention (n=199)	OR^a^/IRR^b^/estimate^c^ (95% CI)	*P* value
**Engagement with SmartQuit app**						
	At least one log-in, n (%)	287 (72)	151 (76)	136 (68)	0.69 (0.44 to 1.07)	.10
	Number of log-ins, mean (SD)	20.54 (34.16)	21.61 (37.74)	19.46 (30.20)	0.90 (0.61 to 1.21)	.38
	Number of days used, mean (SD)	11.96 (16.90)	11.73 (16.14)	12.19 (17.64)	1.00 (0.72 to 1.37)	.97
	Completion certificate, n (%)	119 (30)	58 (30)	61 (31)	1.08 (0.70 to 1.65)	.74
	Number of Acceptance and Commitment Therapy^d^ exercises completed, mean (SD)	11.92 (19.93)	11.74 (17.03)	12.12 (22.50)	0.95 (0.69 to 1.32)	.77
**Smoking cessation**						
	30-day PPA^e^, complete case, n (%)	56 (15)	25 (13)	31 (17)	1.64 (0.90 to 3.00)	.10
	30-day PPA, missing=smoking, n (%)	56 (14)	25 (12)	31 (16)	1.54 (0.86 to 2.76)	.15
	Change in cigarettes per day, mean (SD)	−3.81 (7.88)	−3.01 (8.37)	−4.66 (7.27)	−1.90 (−4.00 to 0.18)	.07
	Change in Fagerström Test for Nicotine Dependence, mean (SD)	−1.33 (1.92)	−1.11 (1.73)	−1.55 (2.08)	−0.53 (−1.07 to 0.01)	.05

^a^OR: odds ratio in logistic regression for binary variables.

^b^IRR: incident rate ratio in negative binomial regression for count variables (ie, number of times logged in and length of use of website).

^c^Point estimate: difference between arms for continuous variables.

^d^Modules inside SmartQuit.

^e^PPA: point prevalence abstinence.

**Table 3:** Smoking cessation and engagement with cessation program at 2-month follow-up with the Modified-Intention-to-Treat analysis. Results are adjusted for 2 stratification factors (heavy smoking and education). Cessation results are adjusted for baseline self-efficacy.

Outcome variable		Overall (N=266)	Control (n=151)	Intervention (n=115)	OR^a^/IRR^b^/estimate^c^ (95% CI)	*P* value
**Engagement with app**						
	Number of log-ins, mean (SD)	30.14 (38.13)	28.48 (41.13)	32.31 (34.00)	1.08 (0.83 to 1.42)	.55
	Number of days used, mean (SD)	17.46 (18.10)	15.46 (16.91)	20.1 (19.31)	4.14 (−0.23 to 8.50)	.06
	Completion certificate, n (%)	116 (44)	58 (38)	58 (50)	1.58 (0.86 to 2.59)	.07
	Number of Acceptance and Commitment Therapy^d^ exercises completed, mean (SD)	17.41 (22.32)	15.48 (18.02)	19.96 (26.82)	3.72 (−1.66 to 9.11)	.18
**Smoking cessation**						
	30-day PPA^e^, complete case, n (%)	42 (17)	19 (13)	23 (21)	2.13 (1.06 to 4.27)	.03
	30-day PPA, missing=smoking, n (%)	42 (16)	19 (13)	23 (20)	2.10 (1.45 to 4.19)	.03
	Change in cigarettes per day, mean (SD)	−4.15 (7.74)	−3.53 (7.57)	−4.96 (7.96)	−1.26 (−3.97 to 1.27)	.33
	Change in Fagerström Test for Nicotine Dependence	−1.48 (1.91)	−1.18 (1.69)	−1.88 (2.12)	−0.78 (−1.45 to −0.11)	.02

^a^OR: odds ratio in logistic regression for binary variables.

^b^IRR: incident rate ratio in negative binomial regression for count variables (ie, number of times logged in and length of use of website).

^c^Point estimate: difference between arms for continuous variables.

^d^Modules inside SmartQuit.

^e^PPA: point prevalence abstinence.

Regarding cessation outcomes, the intervention group participants significantly differed from the control group on quit rates (21% vs 13%) at 2-month follow-up (OR 2.13, 95% CI 1.06-4.27; *P*=.03). The results were the same when missing data were coded as smoking (OR 2.10, 95% CI 1.45-4.19; *P*=.03). For cessation progress, descriptively similar patterns were observed for reduction of smoking, although the difference was not significant (mean decrease in number of cigarettes per day=4.96 vs 3.53; *B*=−1.26 *, P*=.33). The participants in the intervention group also showed greater reduction in nicotine dependence (mean score decrease=1.88 vs 1.18; *B*=−.78; *P*=.02) than the control group.

## Discussion

### Summary of Results

This study evaluated a randomized trial of a growth mindset intervention on engagement with SmartQuit, an app-based cessation program, and successful cessation among daily smokers. On average, participants viewed half of their assigned growth mindset tips in the intervention condition. Contrary to the hypothesis that the growth mindset intervention might improve persistence with the SmartQuit program, there were no significant differences between study arms in engagement with SmartQuit. There was a nonsignificant trend for higher odds of cessation in the intervention group compared with the control group. A modified intention-to-treat analysis was also conducted as a sensitivity analysis to evaluate the impact of including data from participants who never accessed their assigned intervention. In this analysis, there were no significant differences on engagement to SmartQuit, although cessation rates were significantly higher for the MIND intervention group compared with the control group. Thus, the results were similar, but with a stronger signal for efficacy of the MIND intervention.

### Implications for Engagement

Overall, participants in the intervention arm viewed about half of their assigned MIND tips, and about one-fifth of them viewed the entire content provided. A Web-delivered growth mindset intervention, although feasible to implement, did not yield higher log-ins to SmartQuit when compared with the control arm. There are some possible explanations for this. First, the mindset scores were not different between groups at the end of the study (controlling for the baseline mindset), possibly because the growth mindset intervention did not sufficiently increase the growth mindset or participants did not view enough of the growth mindset intervention content to demonstrate a significant overall increase in their mindset score. Given that an overall increase in the growth mindset was hypothesized to improve engagement, this could explain why there were no overall differences in engagement with SmartQuit. To explore this further, a follow-up analysis showed that viewing more MIND tips was significantly associated with increased growth mindset scores in the intervention group (*B*=0.04; *P*=.03). Therefore, future iterations of this work should explore ways to improve the adoption and efficacy of the growth mindset intervention.

Second, it is possible that MIND intervention group participants who received the combination of an app-based and a Web-based intervention might have simply been provided too much content over 2 different modalities such that they did not have time for both. Furthermore, participants in the intervention group may have been responsive to the MIND tips over the app because the emails proactively reached out to them and served as a cue for participation, whereas app use has to be driven by the participant’s own actions. Future work implementing a growth mindset intervention for engagement should take these into consideration for improving on intervention design and delivery. Perhaps, combining all the interventions into a single modality will ease participant burden and improve engagement rates.

In general, there was less engagement across both arms than desired. Although all participants signed up for a technology-based smoking cessation study, more than one-fifth of the sample did not use their assigned programs even once. This study did not require the participants to log in to be enrolled, as imposing that condition would have proven costly and difficult to implement, and our goal was to mimic real-world conditions where participants are free to access their app. Other digital interventions that have not required 1 log-in as an enrollment condition have found that 57% to 82% of the recruited sample did not download/log in to their digital interventions [43,44]. These behaviors may be characteristics of technology-delivered interventions, where factors intrinsic to technology itself (eg, lack of space in a participant’s phone, knowledge needed to install the app, and steps involved in acquiring a password from email to input into a phone) may have affected participant engagement [45]. Although characteristic of mobile health and eHealth studies, the problem of low engagement remains challenging and contributes to difficulties in interpreting study results. Programs using app-based and/or Web-based interventions should carefully consider mitigation strategies for these factors including improving ease of access and early engagement strategies. To address the interpretive issues that arise from failure to engage with any aspect of the intervention, we used a modified intention-to-treat analysis to explore outcomes among users who did engage at least minimally to the treatment protocol. This analysis was intended as a sensitivity analysis, and its conclusions are limited by the absence of random assignment; thus, it cannot be used to draw causal conclusions [46].

Results of the modified intent-to-treat analysis showed no differences in log-ins. Descriptively, participants in the intervention subgroup logged in for a greater number of days and were more likely to achieve the certificate of completion. Half of all participants in the intervention subgroup received a certificate of completion to the SmartQuit program compared with 38% in the control group. Although nonsignificant, the descriptive differences in achieving the certificate of completion are important from a treatment perspective, as previous research has found that smokers who achieved certificate of completion were 4 times more likely to quit. The descriptive difference in results between number of log-ins and number of days logged in also suggests the readers of the mindset tip may have been using the app differently. Additional exploration of the data is needed to examine the nature of this difference (eg, intervention participants logging in to get tips before, during, and after their quit attempt to sustain behavior change instead of consuming large amounts of content at once). Greater number of days logged in may also indicate greater persistence toward behavior change, which would be consistent with the mindset theory.

Another explanation for the differential outcomes is the greater participant exclusion in the experimental subgroup (the experimental subgroup needed at least 1 page view of the MIND content (of 58 total pages of content) and 1 log-in to SmartQuit (relative to the control group, which only needed 1 log-in to SmartQuit), which may have excluded participants who differed in motivation or other variables predisposing them to engagement. However, a post-hoc analysis of the 2 subgroups revealed no significant differences in demographic variables or commitment to quit smoking between them at baseline. There was still a significant difference in baseline self-efficacy to quit between the intervention and control subgroups, but this difference was not related to engagement with SmartQuit. On the basis of available data, then, it is more likely that exposure to a growth mindset intervention is related to the difference between arms than baseline demographic difference. However, we cannot rule out the possibility that other unmeasured variables may have contributed to this difference as well.

### Implications for Cessation

There was a trend toward greater cessation (30-day PPA at 2 months) and reduction in the number of cigarettes smoked per day and in nicotine dependence in the experimental arm compared with the control arm. These results were also mirrored more strongly in the modified intent-to-treat results when examining participants with at least minimal adherence to their assigned interventions. The differences in cessation were present despite the lack of differences in number of log-ins. Reflecting on the fact that mindset interventions most commonly affect behavioral persistence, perhaps a measure of the number of log-ins does not fully capture that variable. A different measure of digital engagement or other external measures that connects more to persistence in behavior toward cessation would be beneficial to investigate in future studies as a mechanism of action.

Viewing all the data on cessation and cessation progress together, there is evidence that the combination of the growth mindset intervention and the SmartQuit app may be more helpful for smokers wanting to quit smoking than the app alone. This result warrants further investigation. When scaled to a population level and considering the cost-effectiveness of technological interventions and health benefits accrued from each additional person who quits or reduces smoking, even a 1% improvement in quit rates can be considered clinically significant [47]. From the results of this study, a 4% improvement (in the intention-to-treat analysis) and an 8% improvement (in the modified intention-to-treat analysis) are considerable when scaled to a population level [47]. Therefore, there is a need to learn from this pilot trial and iterate and improve on the adoption and efficacy of a growth mindset intervention.

### Limitations and Future Directions

This pilot study of a growth mindset intervention has some important limitations to note. The experimental group received a growth mindset intervention in addition to an app, whereas the control group did not receive anything in addition to the app. Future work should explore alternative study designs in addition to the pilot design demonstrated here and compare the growth mindset intervention to other interventions to evaluate its comparative efficacy. Adherence to the MIND Tips remained lower than desired in the intervention arm, and there was no measure of knowledge gained from the MIND Tips included in this study. The sample size limits power to draw inferences on cessation, and pretreatment attrition, although more realistic of real-world use, limits the amount of data available for a full exploration of an intention-to-treat analysis. Although the modified intent-to-treat analysis provides important insight into the outcomes of participants who at least accessed their programs, the selection of participant subgroups cannot be used to draw causal conclusions because of loss of true random assignment [48]. Future studies should focus on making technology-delivered interventions easier to adopt and investigate ways to reduce attrition from participants never engaging with an intervention. Delivering all the intervention content over the same technology may alleviate some burden on participants instead of having tips linked by email and skills delivered through a smartphone app. Future work should also follow up participants for 6 months or more to identify efficacy over the long term. Future studies can also improve on recruiting a diverse sample. Despite targeted enrollment efforts, the study population was still largely white (79%) and had greater than high school education (61%). Finally, future work should also explore the different mechanisms through which the intervention influence engagement and/or cessation, including changes in self-efficacy and commitment to quit smoking.

### Conclusions

A Web-delivered growth mindset intervention for nicotine addiction is feasible to implement but did not enhance engagement with smartphone app for smoking cessation. The combined Web-delivered growth mindset intervention and app may have a beneficial effect for cessation or progress toward cessation. More research is required to improve on the growth mindset intervention, remove barriers to and enhance its adoption, and evaluate its effectiveness in combination with existing cessation programs.

## Appendix

Multimedia Appendix 1 Additional references used to create intervention material.

Multimedia Appendix 2 CONSORT‐EHEALTH checklist (V 1.6.1).

## Abbreviations

AMS: Addiction Mindset Scale
eHealth: electronic health
FTND: Fagerström Test of Nicotine Dependence
IQR: interquartile range
IRR: incident rate ratio
MIND: Mindset Intervention for Nicotine Dependence
OR: odds ratio
PPA: point prevalence abstinence
