# Supplementary material for: A Pilot Randomized Controlled Trial of a Web-Based Growth Mindset Intervention to Enhance the Effectiveness of a Smartphone App for Smoking Cessation
Source: JMIR Mhealth Uhealth. 2019 Jul 9;7(7):e14602. doi: 10.2196/14602 (PMC6647751; doi:10.2196/14602)
Supplement: Multimedia Appendix 1 [file mhealth_v7i7e14602_app1.pdf]

Supplemental Material – Literature review used to develop content for MIND intervention

1. Mamede M, Ishizu K, Ueda M, et al. Temporal change in human nicotinic acetylcholine receptor after smoking cessation: 5IA SPECT study. *J Nucl Med*. 2007;48(11):1829-1835.
2. Cosgrove KP, Batis J, Bois F, et al. beta2-Nicotinic acetylcholine receptor availability during acute and prolonged abstinence from tobacco smoking. *Arch Gen Psychiatry*. 2009;66(6):666-76.
3. Jasinska AJ, Zorick T, Brody AL, Stein EA. Dual role of nicotine in addiction and cognition: A review of neuroimaging studies in humans. *Neuropharmacology*. 2014. doi:10.1016/j.neuropharm.2013.02.015.
4. O'Connell KA, Gerkovich MM, Cook MR, Shiffman S, Hickcox M, Kakolewski KE. Coping in Real Time: Using Ecological Momentary Assessment Techniques to Assess Coping with the Urge to Smoke. *Res Nurs Heal*. 1998. doi:10.1002/(SICI)1098-240X(199812)21:6<487::AID-NUR3>3.0.CO;2-G.
5. Center for Disease Control. (2017) Managing Withdrawal. Retrieved from https://www.cdc.gov/tobacco/campaign/tips/quit-smoking/guide/withdrawal.html
6. Hall WD, Gartner CE, Carter A. The genetics of nicotine addiction liability: Ethical and social policy implications. *Addiction*. 2008;103(3):350-359. doi:10.1111/j.1360-0443.2007.02070.x.
7. Furberg H, Kim Y, Dackor J, et al. Genome-wide meta-analyses identify multiple loci associated with smoking behavior. *Nat Genet*. 2010. doi:10.1038/ng.571.
8. Liu JZ, Tozzi F, Waterworth DM, et al. Meta-analysis and imputation refines the association of 15q25 with smoking quantity. *Nat Genet*. 2010. doi:10.1038/ng.572.
9. Amodeo M. The Addictive Personality. *Subst Use Misuse*. 2015. doi:10.3109/10826084.2015.1007646.
10. Szalavitz, M. (2015). No more addictive personality. *Nature*, 522, S48–S49. http://doi.org/10.1038/522S48a
11. Satel S, Lilienfeld SO. Addiction and the brain-disease fallacy. *Front Psychiatry*. 2014. doi:10.3389/fpsyt.2013.00141.
12. Hughes JR. Craving among long-abstinent smokers: an Internet survey. *Nicotine Tob Res*. 2010;12(4):459-62.
13. National Institute on Drug Abuse .https://easyread.drugabuse.gov/content/what-addiction. Accessed 26 June 2016.
14. Goldstein RZ, Volkow ND. Drug addiction and its underlying neurobiological basis: Neuroimaging evidence for the involvement of the frontal cortex. *Am J Psychiatry*. 2002. doi:10.1176/appi.ajp.159.10.1642.
15. Stevenson JG, Oliver JA, Hallyburton MB, Sweitzer MM, Conklin CA, McClernon FJ. Smoking environment cues reduce ability to resist smoking as measured by a delay to smoking task. *Addict Behav*. 2017. doi:10.1016/j.addbeh.2016.12.007.

16. Centers for Disease Control and Prevention. https://www.cdc.gov/tobacco/data_statistics/fact_sheets/cessation/quitting/. Accessed November 14, 2016.
17. Centers for Disease Control and Prevention. Cigarette Smoking Among Adults—United States, 2005–2015. Morbidity and Mortality Weekly Report 2016;65(44):1205–11 [accessed 2016 Nov 14].)
18. US Department of Health and Human Services. The Health Consequences of Smoking—50 Years of Progress A Report of the Surgeon General. *A Rep Surg Gen*. 2014. doi:NBK179276.
19. Hughes JR, Peters EN, Naud S. Relapse to smoking after 1 year of abstinence: A meta-analysis. *Addict Behav*. 2008. doi:10.1016/j.addbeh.2008.05.012.
20. Hawkins J, Hollingworth W, Campbell R. Long-Term smoking relapse: A study using the British Household Panel Survey. *Nicotine Tob Res*. 2010. doi:10.1093/ntr/ntq175.
21. Hajek P, Stead LF, West R, Jarvis M, Hartmann-Boyce J, Lancaster T. Relapse prevention interventions for smoking cessation. *Cochrane Database Syst Rev*. 2013. doi:10.1002/14651858.CD003999.pub4.
22. Chaiton M, Diemert L, Cohen JE, et al. Estimating the number of quit attempts it takes to quit smoking successfully in a longitudinal cohort of smokers. *BMJ Open*. 2016;6(6):e011045. Published 2016 Jun 9. doi:10.1136/bmjopen-2016-011045
